# Verbal and non-verbal memory and hippocampal volumes in a memory clinic population

**DOI:** 10.1186/s13195-015-0147-9

**Published:** 2015-10-15

**Authors:** Aaron Bonner-Jackson, Shamseldeen Mahmoud, Justin Miller, Sarah J Banks

**Affiliations:** Lou Ruvo Center for Brain Health, Neurological Institute, Cleveland Clinic, 9500 Euclid Avenue/U10, Cleveland, OH 44195 USA; Lou Ruvo Center for Brain Health, Neurological Institute, Cleveland Clinic, Las Vegas, NV USA

## Abstract

**Introduction:**

Better characterization of the relationship between episodic memory and hippocampal volumes is crucial in early detection of neurodegenerative disease. We examined these relationships in a memory clinic population.

**Methods:**

Participants (*n* = 226) underwent structural magnetic resonance imaging and tests of verbal (Hopkins Verbal Learning Test-Revised, HVLT-R) and non-verbal (Brief Visuospatial Memory Test-Revised, BVMT-R) memory. Correlational analyses were performed, and analyses on clinical subgroups (i.e., amnestic Mild Cognitive Impairment, non-amnestic Mild Cognitive Impairment, probable Alzheimer’s disease, intact memory) were conducted.

**Results:**

Positive associations were identified between bilateral hippocampal volumes and both memory measures, and BVMT-R learning slope was more strongly positively associated with hippocampal volumes than HVLT-R learning slope. Amnestic Mild Cognitive Impairment (aMCI) participants showed specific positive associations between BVMT-R performance and hippocampal volumes bilaterally. Additionally, analyses of the aMCI group showed trend-level evidence of material-specific lateralization, such that retention of verbal information was positively associated with left hippocampal volume, whereas learning curve and retention of non-verbal information was positively associated with right hippocampal volume.

**Conclusions:**

Findings support the link between episodic memory and hippocampal volumes in a memory clinic population. Non-verbal memory measures also may have higher diagnostic value, particularly in individuals at elevated risk for Alzheimer’s disease.

## Introduction

Medial temporal lobe (MTL) structures (e.g., hippocampus) are integral to the formation of new memories and are centrally related to development of Alzheimer’s disease (AD) [1]. Specifically, MTL atrophy and associated episodic memory impairment are hallmark features of AD, and both progressively decline over the course of the disease [2, 3].

Material-specific lateralization of brain function is a classic neurological and neuropsychological finding [4]. Lesion and functional neuroimaging studies have found that memory function is lateralized based on material type [5] (see [6] for review). In general, left hemisphere structures (including left hippocampus) are implicated in verbal memory processing [7], while right hemisphere structures (including right hippocampus) support non-verbal/spatial memory [8].

Additionally, some previous work using various neuroimaging methodologies (e.g., voxel-based morphometry) suggests positive associations between hippocampal size and memory performance [9–12], including in patients with AD [13–21]. However, there is significant variability among older adults with regard to this relationship, with some groups finding no evidence of an association [22] (see [23] for review). Better characterization of the relationship between commonly used episodic memory measures and hippocampal volumes is crucial, as it may assist in efforts at early detection of neurodegenerative disease and may suggest new biomarkers for use in clinical trials of AD therapies.

Previous studies in this area have had limitations. De Toledo-Morrell et al. [24] examined the relationship between hippocampal volumes and verbal vs. non-verbal memory processing in individuals with probable AD and healthy older adults. They found that left hippocampal volume was the strongest predictor of verbal memory, whereas right hippocampal volume was the strongest predictor of spatial memory. Similar findings were reported by Kohler and colleagues [15] in individuals with AD. However, our study has various advantages over previous work that allow for more accurate acquisition and segmentation of brain images and better characterization of memory performance. For example, Kohler and colleagues used a 1.5T Signa system with a standard head coil to acquire brain images. Volumes of interest were determined using ANALYZE software, and manual tracing ("planimetry") was used to delineate the hippocampus. In contrast, our study used fully automated FDA-approved software for hippocampal segmentation. We used the Alzheimer's Disease Neuroimaging Initiative (ADNI) sequence, and 3 Tesla MRI scanners were utilized for imaging in all subjects. Furthermore, most previous studies have lacked inclusion of other groups of interest, including individuals with Mild Cognitive Impairment (MCI) who are at elevated risk for development of AD. This is a particularly crucial point, as the AD disease process is hypothesized to begin years before symptom onset, and evidence of significant MTL atrophy has been found even in those at the MCI stage [25, 26].

Although verbal memory tasks often have been more commonly utilized (e.g., [19, 27, 28], there is increasing support for the role of non-verbal memory tasks in dementia assessment. Various studies (i.e., [29, 30] have reported that rate of learning on non-verbal memory measures has better diagnostic value than verbal memory measures and can discriminate individuals at various stages (i.e., controls vs. MCI vs. mild AD). Okonkwo et al. [31] found that non-verbal learning and delayed recall performance among cognitively-intact older adults significantly dissociated individuals who showed cognitive decline over a follow-up period from those who remained cognitively stable. Others have found that visual, rather than verbal, memory measures were predictive of memory decline and development of AD [32, 33]. As such, further examination of the relative merits and predictive value of these measures is warranted, particularly as they relate to volumes of the hippocampus.

The current study examined the relationships between hippocampal volumes (as measured by NeuroQuant software) and performance on two analogous measures of verbal and non-verbal episodic memory (i.e., Hopkins Verbal Learning Test-Revised [34], Brief Visuospatial Memory Test-Revised [35]) in a sample of memory clinic patients. We had three objectives: 1) explore whether episodic memory performance is related to hippocampal volumes in our sample; 2) determine whether associations are lateralized in a material-specific manner (i.e., verbal memory performance more strongly related to left hippocampal volume, non-verbal memory performance more strongly related to right hippocampal volume); and 3) examine these relationships in diagnostic groups of interest who may be at higher risk for development of dementia (i.e., amnestic MCI). We predicted significant positive relationships between hippocampal volumes and memory measures for the whole sample, as well as material-specific lateralization of relationships between hippocampal volumes and memory performance. Additionally, we predicted similar relationships in individuals with amnestic MCI and AD.

## Methods

### Participants

Archival clinical data were extracted from the electronic medical records of patients seen for clinical exams in the Center for Brain Health at the Cleveland Clinic (Cleveland, OH, and Las Vegas, NV) in accordance with regulations of the Institutional Review Board of the Cleveland Clinic, and the nature of data collection indicated that no informed consent was required of participants. Participants (*n* = 226) were included in the study if they had undergone structural magnetic resonance imaging (MRI) of the brain (for quantification of hippocampal volumes) and neuropsychological evaluation in the course of routine clinical care. Demographic and descriptive information is presented in Table 1. Diagnoses were determined through consensus conference or consultation with the referring physician and included patients with Alzheimer’s disease (*n* = 34), Mild Cognitive Impairment, amnestic type (*n* = 82), Mild Cognitive Impairment, non-amnestic type (*n* = 13) and age-associated memory changes (i.e., normal memory; *n* = 25). The remainder of the sample (*n* = 72) consisted of patients with other neurological disorders typically seen in a dementia clinic, primarily those with neurodegenerative disorders (e.g., frontotemporal dementia, primary progressive aphasia, corticobasal syndrome, etc.), as well as a small percentage diagnosed with stroke or vascular dementia.

**Table 1:** Demographic and behavioral data

	Entire sample	aMCI	naMCI	AD	Normal memory
	(*N* = 226)	(*N* = 86)	(*N* = 13)	(*N* = 34)	(*N* = 25)
Age (yrs)^a^	67.7 (9.1)	67.8 (8.4)	67.7 (6.6)	71.2 (9.8)	63.6 (5.8)
Sex (% female)	57.1	59.8	53.8	58.8	56.0
Education (yrs)	14.5 (2.9)	14.8 (2.7)	13.7 (2.4)	14.7 (3.1)	15.5 (2.9)
Handedness (% right)	89.8	85.4	92.3	91.2	88.0
HVLT-R Learning^b^	17.2 (6.6)	16.3 (5.3)	21.3 (4.8)	11.7 (4.1)	25.5 (3.3)
HVLT-R Recall^c^	3.9 (3.8)	2.5 (2.8)	7.2 (2.1)	1.0 (1.7)	9.6 (1.6)
BVMT-R Learning^d^	11.4 (7.8)	9.7 (5.8)	18.9 (6.4)	5.5 (3.7)	22.0 (4.4)
BVMT-R Recall^e^	4.1 (3.5)	3.3 (2.9)	7.7 (2.5)	1.5 (1.8)	8.8 (1.9)
R Hippocampus (cm^3^)^f^	3.7 (0.6)	3.7 (0.6)	4.0 (0.4)	3.3 (0.6)	4.1 (0.4)
L Hippocampus (cm^3^)^g^	3.5 (0.5)	3.5 (0.5)	3.6 (0.4)	3.1 (0.5)	3.8 (0.4)

Values represent means unless otherwise indicated. Standard deviations presented in parentheses

*aMCI* Mild Cognitive Impairment, amnestic type, *naMCI* Mild Cognitive Impairment, non-amnestic type, *AD* Alzheimer’s disease, *HVLT-R* Hopkins Verbal Learning Test-Revised, raw score, *BVMT-R* Brief Visuospatial Memory Test-Revised, raw score

^a^AD > Normal Memory (*p* < .005).

^b^Normal Memory > non-amnestic MCI > amnestic MCI > AD

^c^Normal Memory > non-amnestic MCI > amnestic MCI > AD

^d^Normal Memory = non-amnestic MCI > amnestic MCI > AD

^e^Normal Memory = non-amnestic MCI > amnestic MCI > AD

^f^Normal Memory = non-amnestic MCI > amnestic MCI > AD

^g^Normal Memory > amnestic MCI = non-amnestic MCI > AD

### Neuropsychological measures

We used performance on two episodic memory measures as variables of interest: the Hopkins Verbal Learning Test-Revised (HVLT-R; [34]) and the Brief Visuospatial Memory Test-Revised (BVMT-R; [35]).

The HVLT-R is a 12-item verbal episodic memory measure in which words are read aloud over three learning trials. Following each presentation of the word list, the participant recites as many words as can be recalled spontaneously, and the total number of words recalled over the three learning trials is recorded (range = 0–36). Following a 20-minute delay, the participant spontaneously recalls as many words as possible. Lastly, a recognition trial is presented in which the participant is required to discriminate the previously-presented words from new foil words.

The BVMT-R is a non-verbal episodic memory measure. Test stimuli consist of six geometric shapes presented over three learning trials. The participant is presented with the array of six shapes for 10 seconds each. Following each of the three learning trials, the participant is asked to spontaneously reproduce as many shapes as possible. Each shape is worth a maximum of two points (one point for correct form, one point for correct location), and total learning over the three trials is measured (range = 0–36). Delayed recall and recognition are also measured. The overall structure of the measure is similar to the HVLT-R, and the two measures are co-normed.

### Structural MRI parameters

Volumetric analysis of the brain was performed on a separate workstation for all participants following acquisition of the structural MRI. Software-compatible high resolution T1-weighted anatomical MRI scans were obtained on all patients, (T1-weighted 3D Sagittal MP-RAGE). ADNI (Alzheimer's Disease Neuroimaging Initiative) protocol was utilized for all studies performed starting 7 January 2010. Prior to this date, axial 3-D T1 MPRAGE was used on a small number of participants included in this study. Participants were scanned using four scanner models, all compatible with the analysis software. Acquisition protocol details are as follows: TR/TE/TI = 2300/2.98/900, Flip Angle = 9, BW = 240 H z/Px, 240 × 256 matrix, 160 slices, voxel size =1 × 1 × 1.2 mm, scan time: 9:14. The scanners are detailed as follows: A Siemens 3T Verio scanner with a 12-channel head coil (Siemens Medical Systems, Erlangen, Germany), Siemens 3T Skyra scanners with a 16 channel head coil (Siemens Medical Systems, Erlangen, Germany), Siemens 3T Trio scanners with a 12 channel head coil (Siemens Medical Systems, Erlangen, Germany), and a Siemens 1.5T Symphony scanner for patients with any contraindication to 3T scanning.

**Fig. 1:**
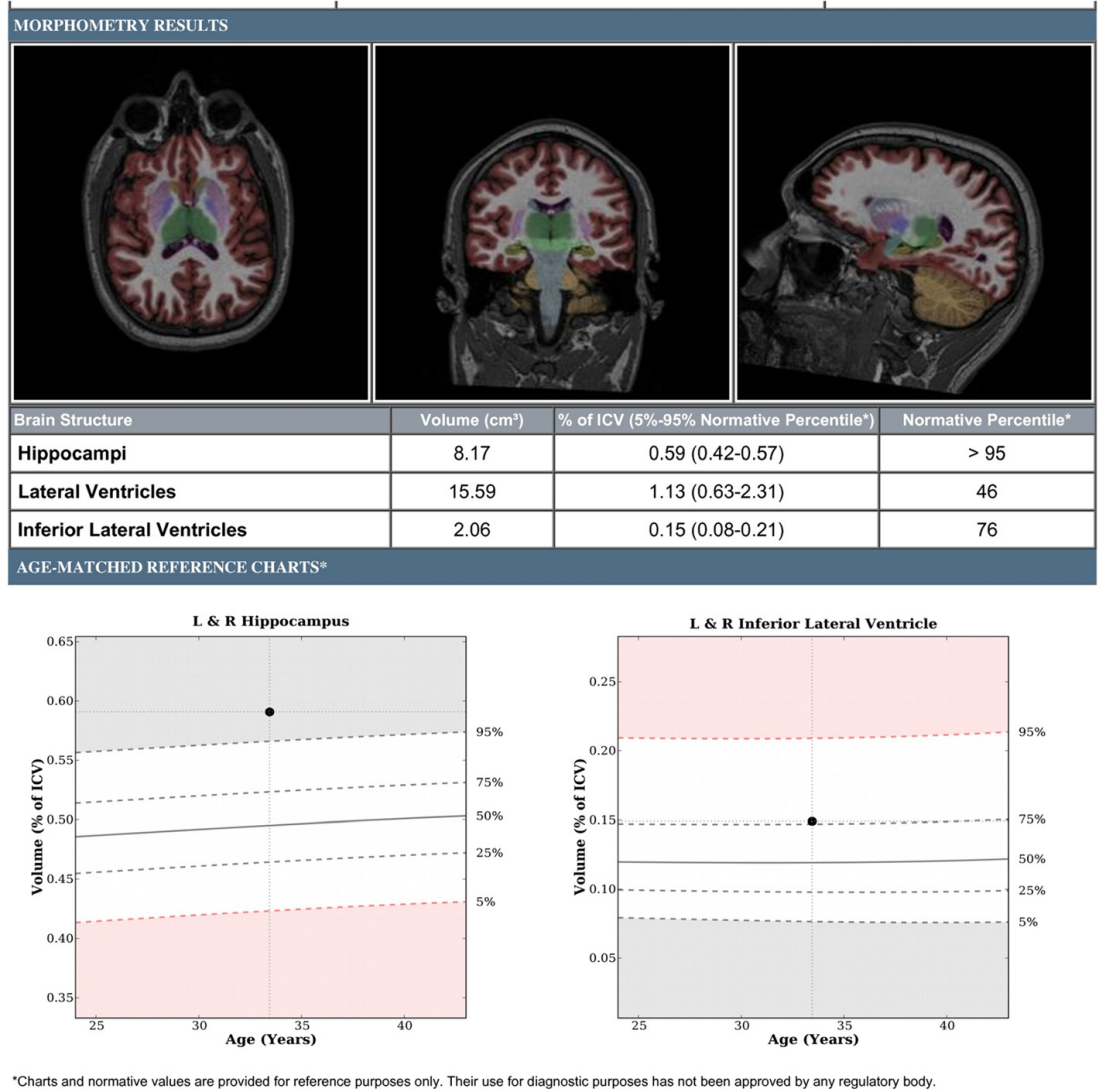
Example output of the NeuroQuant computer-automated analysis, including volumetric information and segmented brain images

Following acquisition, images from the ADNI sequence were sent to the image analysis lab at the Cleveland Clinic for volumetric analysis. NeuroQuant software (CorTechs Labs, Inc., San Diego, CA, USA) was used for analysis. NeuroQuant is a commercially available FDA-approved software program for measuring brain MRI volume in clinical settings. Images were sent directly from the scanners to the NeuroQuant server where analysis started automatically and results were routed automatically to the Radiology Archives, Cleveland Clinic. Initially, images were sent to open-source intermediate viewing software (OsirX), then to the NeuroQuant server. NeuroQuant analysis involved several automated steps, including stripping the brain of scalp, skull and meninges; inflating the brain to a spherical shape; mapping the spherical brain to a common spherical space shared with the Talairach Atlas brain [36]; identification of brain regions; and deflation of the patient’s brain back to its original shape while retaining the identifying information for brain segments. The output of the NeuroQuant computer-automated analysis included a report containing volumetric information, and a set of DICOM-formatted brain images that were segmented, with each region identified by a distinctive color [37] (see Fig. 1).

Visual inspection of the anatomical images was performed to ensure good image quality and absence of notable artifacts. Additionally, review of the color-coded images was performed for each subject to ensure quality of hippocampal segmentation. Hippocampal segmentation quality was graded as excellent, adequate or poor, depending on the presence and degree of any segmentation errors. Failure of analysis (in rare instances) was due to faulty registration, either due to severe motion or severe volume loss (marked ventricular dilatation). Subjective assessment of segmentation quality was used by visual inspection of the color coded images for color mismatch.

**Fig. 2:**
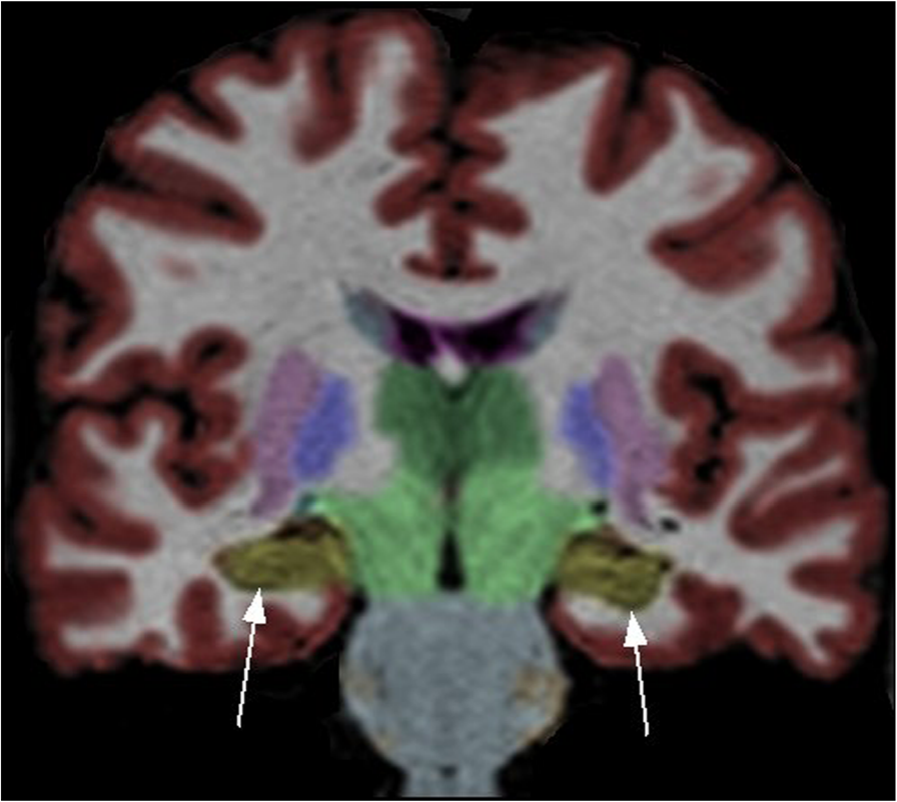
Coronal image of NeuroQuant output, with arrows pointing to the hippocampus

Although volumetric values of various brain regions were calculated, only volumes of the right and left hippocampus were extracted for the purposes of these analyses (see Fig. 2).

In-house developed software was used to calculate the total brain volume and percent of the intracranial volume. Intracranial volume was calculated based on total brain volume and hippocampal volume as a percentage of total intracranial volume. Certain volumetric measures were included in the clinical reports for all participants. A statement about study and segmentation qualities was included in the report. Quality was graded subjectively as adequate, marginal, or poor. All data were pooled into the Cleveland Clinic’s Knowledge Project database, among other clinical and neuropsychological data, where pertinent data were retrieved according to the study protocol.

### Data analysis

Data were analyzed to examine relationships between performance on the episodic memory measures and hippocampal volumes. Partial correlations were conducted to account for patient age, patient sex, and total intracranial volume, as both factors are significantly associated with hippocampal volumes. Fisher’s r-to-z transformations were used to compare magnitudes of correlations (e.g., across hemisphere, across clinical groups, etc.). Initial analyses included the entire participant sample, while subsequent analyses targeted individual diagnostic groups (i.e., amnestic MCI, AD). Variables of interest for the memory measures included performance on individual learning trials, total three-trial learning, learning over trials (i.e., difference between Trial 1 and Trial 3), delayed recall, and percent retention (i.e., Delayed Recall/Trial 3).

## Results

### Demographic, behavioral, and volumetric data

Descriptive data for demographic, behavioral, and volumetric variables are presented in Table 1. Among the specific diagnostic groups, the AD group was significantly older than the Normal Memory group. No other between-group differences were found for age or any of the other demographic variables (i.e., education, sex, handedness).

Next, diagnostic groups were compared to assess for between-group differences on the behavioral variables. Results of one-way analyses of variance (ANOVAs) indicated significant between-group differences on all behavioral variables (all p’s < .001), and post-hoc comparisons were conducted. On both HVLT-R Learning and HVLT-R Recall, the Normal Memory group performed significantly better than all other diagnostic groups, and the non-amnestic MCI group performed better than the amnestic MCI and AD groups, while the amnestic MCI group performed better than only the AD group. On both BVMT-R Learning and BVMT-R Recall, the Normal Memory and non-amnestic MCI groups did not significantly differ from each other, while both performed better than the amnestic MCI and AD groups. In turn, the amnestic MCI group performed significantly better than the AD group.

Diagnostic groups were then compared to assess for between-group differences in hippocampal volumes. Results of one-way ANOVAs indicated significant between-group differences in both left and right hippocampal volumes (p’s < .001). For right hippocampus, post-hoc comparisons revealed that the Normal Memory and non-amnestic MCI groups were equivalent, and both had larger volumes than the amnestic MCI and AD groups. In turn, the amnestic MCI group had larger volumes than the AD group. For left hippocampus, the Normal Memory and non-amnestic groups did not significantly differ from each other. The Normal Memory group had larger volumes than the amnestic MCI group, while the amnestic MCI and non-amnestic MCI groups did not differ, and all groups had larger volumes than the AD group.

Tests of normality revealed that many of the behavioral and volumetric variables were not normally distributed (i.e., HVLT-R Delayed Recall, BVMT-R Learning, BVMT-R Delayed Recall, HVLT-R Retention and Learning over Trials, BVMT-R Retention and Learning over Trials, right hippocampal volumes). As such, we conducted log transformations on all behavioral and hippocampal volume data, and the analyses on the transformed variables are presented below.

### Correlational analyses – entire sample

#### Total learning and delayed recall

Significant positive correlations were found between learning and recall on both HVLT-R and BVMT-R and volumes of left and right hippocampus (Table 2). Using Fisher’s r-to-z transformation to compare the correlations, we found that the magnitude of the correlations did not differ as a function of hemisphere.

**Table 2:** Correlations between learning and recall performance and hippocampal volumes for the entire sample

	HVLT-R – Total learning	HVLT – Recall	BVMT-R – Total learning	BVMT-R – Recall
R Hippocampus	.232*	.302**	.278**	.343**
L Hippocampus	.326**	.316**	.289**	.328**

*BVMT-R* Brief Visuospatial Memory Test-Revised, *HVLT-R* Hopkins Verbal Learning Test-Revised

*p < .005

**p < .001

#### Learning over trials

Correlations between hippocampal volumes and learning over trials indicated a specific positive relationship between non-verbal learning and hippocampal volumes (Table 3). Specifically, learning over trials on the BVMT-R was significantly positively associated with volumes of both left and right hippocampus. In contrast, no such relationships were found between the HVLT-R and left or right hippocampus, although the relationship between HVLT-R learning over trials and right hippocampal volumes reached trend-level significance (*p* = .06). Again, the magnitude of the correlations did not differ as a function of hemisphere.

**Table 3:** Correlations between learning over trials and retention and hippocampal volumes for the entire sample

	HVLT-R – LOT	HVLT-R – Retention	BVMT-R – LOT	BVMT-R - Retention
R Hippocampus	.13	.30**	.23**	.23*
L Hippocampus	.11	.29**	.22*	.23*

*HVLT-R*, Hopkins Verbal Learning Test-Revised, *LOT* Learning over trials (i.e., Trial 3 – Trial 1), *BVMT-R* Brief Visuospatial Memory Test-Revised

Retention = Percent of information initially learned that is spontaneously recalled at Delayed Recall (i.e., Delay/Trial 3)

**p < .001

*p < .005

#### Retention

Associations between retention over delay periods and hippocampal volumes were assessed next (Table 3). Percent retention for the HVLT-R was significantly positively associated with volume of hippocampus bilaterally. Similarly, significant positive associations were found between BVMT-R retention and hippocampal volumes bilaterally. The magnitudes of the correlations did not differ as a function of hemisphere.

### Correlational analyses – amnestic MCI group

#### Total learning and delayed recall

We next analyzed the data separately for the amnestic MCI group (*n* = 82). We found evidence for a specific positive relationship between non-verbal memory performance and hippocampal volumes in this group (Table 4). Specifically, we detected significant positive correlations between BVMT-R total learning and delayed recall and hippocampal volumes bilaterally. The magnitudes of the correlations did not significantly differ by hemisphere. In contrast, no significant associations were detected between HVLT-R total learning or delayed recall and hippocampal volumes. Examination of the correlation coefficients revealed that the relationship between BVMT-R total learning and right hippocampal volume was significantly higher than the relationship between HVLT-R total learning and right hippocampal volume (*p* < .05). No other correlation coefficients differed significantly across tasks.

**Table 4:** Correlations between learning and recall performance and hippocampal volumes for the amnestic MCI group

	HVLT-R – Total learning	HVLT-R – Recall	BVMT-R – Total learning	BVMT-R - Recall
R Hippocampus	.01	.12	.34***	.30**
L Hippocampus	.10	.19	.27*	.23*

*MCI* Mild cognitive impairment, *HVLT-R*, Hopkins Verbal Learning Test-Revised, *BVMT-R* Brief Visuospatial Memory Test-Revised

***p < .005

**p < .01

*p < .05

#### Learning over trials and retention

We next investigated the relationship between hippocampal volumes and both learning over trials and percent retention on both memory measures (Table 5). None of the correlations reached statistical significance. However, the relationships between right hippocampal volume and both BVMT-R learning over trials (*p* = .075) and BVMT-R retention (*p* = .056) were significant at a trend level. The relationship between HVLT-R retention and left hippocampal volume also reached trend-level significance (*p* = .063).

**Table 5:** Correlations between learning over trials and retention and hippocampal volumes for the amnestic MCI group

	HVLT-R – LOT	HVLT-R – Retention	BVMT-R – LOT	BVMT-R - Retention
R Hippocampus	-.03	.17	.20	.22
L Hippocampus	-.02	.22	.09	.20

*MCI* Mild cognitive impairment, *HVLT-R*, Hopkins Verbal Learning Test-Revised, *LOT* Learning over trials (i.e., Trial 3 – Trial 1), *BVMT-R* Brief Visuospatial Memory Test-Revised

Retention = Percent of information initially learned that is spontaneously recalled at Delayed Recall (i.e., Delay/Trial 3)

### Correlational analyses – additional subgroups

For purposes of comparison, we examined a subgroup of patients with intact memory performance (*n* = 25), as well as patients characterized as non-amnestic Mild Cognitive Impairment (*n* = 13). We found that none of the correlations between learning or recall measures and hippocampal volumes reached significance for either BVMT-R or HVLT-R in either group. In fact, many of the associations were negative.

We also examined a subgroup of patients diagnosed with probable Alzheimer’s disease (*n* = 34). Again, none of the correlations between memory measures and hippocampal volumes reached significance.

## Discussion

In the current study, we examined associations between hippocampal volumes and memory performance in a large, heterogeneous memory clinic sample, using commonly-used memory measures and an FDA-approved automated neuroimaging analysis tool. Within this context, there were a number of main findings. The sample as a whole showed significant positive correlations between bilateral hippocampal volumes and both verbal and non-verbal memory measures. Additionally, the BVMT-R showed more positive associations with hippocampal volumes than indices on the HVLT-R. In the amnestic MCI group, we found further evidence of a specific positive association between non-verbal memory performance and hippocampal volumes bilaterally. Additionally, we found evidence of material-specific (i.e., verbal vs. non-verbal) lateralization in the amnestic MCI group, although these findings did not reach statistical significance. We describe each of these main findings below.

### Entire sample

The full sample showed significant positive associations between hippocampal volumes and performance on episodic memory measures. Consistent with previous work in this area [9–12, 18], indices of memory performance (i.e., total learning, percent retention, and delayed recall) on both the verbal and non-verbal measures were significantly positively correlated with volumes of hippocampus bilaterally. These findings confirm the association between hippocampal integrity and performance on episodic memory measures typically administered in an outpatient memory clinic.

Contrary to our predictions, we did not find evidence of material-specific lateralization with regard to relationships between hippocampal volumes and memory performance, as the strength of the relationships did not differ as a function of hemisphere. The reasons for this are unclear, as some researchers who have examined this question in individuals with AD have reported lateralization effects [15, 24]. It is possible that analysis of the full heterogeneous sample (rather than just the AD group) affected the outcome, although our analysis of the AD patients alone did not yield evidence of lateralization. Second, the differences in findings across studies also may reflect differences in the memory tasks used, as the tasks in the current study did not overlap with those in which lateralization effects were found. Lastly, one such study [15] found laterality effects in the right parahippocampal gyrus, whereas we restricted our analyses to the hippocampus. Thus, it is possible that we would have detected laterality effects had we examined more structures in the mesial temporal lobes.

Across the whole sample, therefore, hippocampal volumes showed significant positive relationships with memory measures in both modalities. However, learning over trials on the BVMT-R was significantly positively associated with volumes of both left and right hippocampus, while no such relationship was detected for the HVLT-R. Although the magnitudes of the correlations did not significantly differ, non-verbal learning over trials (or “learning curve”) appears to be somewhat more sensitive to hippocampal integrity in a manner that is not seen as prominently for verbal learning.

Previous studies of MCI and AD generally have emphasized deficits in verbal learning [38–41], and associations between verbal episodic memory performance and hippocampal volumes in individuals with MCI have been reported [42]. However, the current findings indicate that examination of the learning curve for non-verbal information also should be considered and may provide valuable information regarding potential degenerative changes. In accord with our findings, increasing evidence suggests a role for non-verbal memory measures, including rate of learning, in differential diagnosis of older adults with memory dysfunction [29, 30], as well as prediction of decline and development of AD [31–33]. Our results extend these findings by demonstrating an association between non-verbal memory (including learning over trials) and a commonly-used biomarker of AD. Relatedly, most clinical trials of AD therapies have used only verbal measures in assessment of memory. Our findings suggest that inclusion of non-verbal memory measures will be an important consideration for future trials. It is possible that inherent properties of BVMT-R make it a strong tool in measuring memory function in a dementia clinic setting. For example, participants are scored on both accuracy of form and accuracy of location of their drawings. As such, the emphasis on spatial memory and the necessity of binding form with location may make the BVMT-R a more rigorous measure of hippocampal integrity than verbal measures.

### Amnestic MCI subgroup

Similar to the whole-group findings, analyses among individuals with amnestic MCI revealed significant positive associations between bilateral hippocampal volumes and both total learning and delayed recall on BVMT-R, while no such relationships were detected for the HVLT-R. Additionally, the correlation between BVMT-R total learning and right hippocampal volume was significantly larger than the correlation between HVLT-R total learning and right hippocampal volume. These results highlight the utility of non-verbal memory measures in dementia assessment, particularly for those at the MCI stage. To our knowledge, few (if any) studies have examined this issue in those with amnestic MCI, which represents a high-risk group for development of AD. This finding also has significant clinical implications, as it provides useful information regarding neuropsychological measures that may best predict conversion to AD in those with MCI.

Additional analysis of the data for the amnestic MCI group revealed trend-level evidence of material-specific lateralization. Specifically, we found a positive association between left hippocampal volumes and HVLT-R retention (p = .063), as well as positive relationships between right hippocampal volumes and BVMT-R learning curve (p = .075) and retention (p = .056). While not statistically significant, our findings somewhat resemble those of Jones-Gotman [43], who found that left MTL seizures were associated with impaired verbal recall, while right MTL seizures were associated with impaired non-verbal learning. Kennepohl and colleagues [44] reported similar findings among healthy control participants. This finding may suggest a division of labor within the MTL with regard to memory processes and supports the value of both verbal and non-verbal memory measures in the assessment of those at risk for AD. As noted above, however, these findings did not reach statistical significance and should be interpreted cautiously. Future studies are required to further evaluate this issue in individuals with amnestic MCI.

Given previous findings in the literature, it was somewhat surprising that no significant associations were detected in the probable AD group, although lack of power due to smaller sample size may have contributed to this finding. Restriction of range in hippocampal size and/or memory performance also may have limited our ability to detect significant relationships in the AD group. Nevertheless, these results suggest an important role for non-verbal memory measures in assessing individuals with amnestic MCI.

Overall, strengths of the current study include use of a large, heterogeneous sample of patients typically seen in a dementia clinic within an academic medical center. Our patients were well-characterized with regard to cognitive performance and underwent full neuropsychological batteries, including memory measures that are commonly used in dementia clinics and are co-normed with each. Neuroimaging data were analyzed with FDA-approved software (NeuroQuant) that automatically segmented and quantified hippocampal volumes. Samples of this size that include both neuroimaging and thorough cognitive evaluation are rare in studies in this area. Additionally, we were able to include a large subset of individuals with amnestic MCI to examine those who are at elevated risk for development of AD.

### Limitations

As mentioned previously, smaller sample sizes in the other diagnostic groups, relative to the amnestic MCI group, may have limited our ability to find significant associations. Replication of these findings with larger sample sizes is, therefore, warranted. Use of multiple correlational analyses also increased our risk of Type I (i.e., false positive) errors. However, the vast majority of associations showed moderate effect sizes, reducing the likelihood that they were spurious. Additionally, it should be noted that output of the NeuroQuant program includes volumes of many other brain structures (e.g., thalamus, amygdala, caudate, putamen, etc.). However, given its central role in the development of Alzheimer’s disease, we chose to restrict our analyses only to the hippocampus. Examination of the relationship between volumes of other brain regions and episodic memory performance will be an area of future study.

## Conclusions

Taken together, our findings indicate significant positive associations between volumes of the hippocampus bilaterally and memory function in our memory clinic sample, with evidence to suggest that the learning curve on a non-verbal memory measure represents a key marker of hippocampal integrity. Among individuals with amnestic MCI, a specific association was identified between non-verbal learning and recall performance and bilateral hippocampal volumes. We also found trend-level evidence of material-specific lateralization, such that retention of verbal information was positively associated with left hippocampal volume, whereas learning curve and retention of non-verbal information was positively associated with right hippocampal volume.

There is increasing consensus supporting the use of multiple markers, including neuropsychological measures and hippocampal volumes, in diagnosis and prediction of disease course in neurodegenerative disorders [26, 45–47]. We provide evidence to support the link between performance on episodic memory measures and hippocampal volumes in a memory clinic population, and we assert that measures of non-verbal memory may have higher diagnostic value, particularly in individuals at higher risk for development of AD (i.e., amnestic MCI), which represents a target for future research.

## Abbreviations

AD: Alzheimer’s disease
ADNI: Alzheimer’s Disease Neuroimaging Initiative
BVMT-R: Brief Visuospatial Memory Test-Revised
HVLT-R: Hopkins Verbal Learning Test-Revised
MCI: Mild cognitive impairment
MRI: Magnetic resonance imaging
MTL: Medial temporal lobe
